# A flexible, thin-film microchannel electrode array device for selective subdiaphragmatic vagus nerve recording

**DOI:** 10.1038/s41378-023-00637-6

**Published:** 2024-01-23

**Authors:** Jongcheon Lim, Peter A. Zoss, Terry L. Powley, Hyowon Lee, Matthew P. Ward

**Affiliations:** 1 Weldon School of Biomedical Engineering, Purdue University, West Lafayette, IN USA; 2 Birck Nanotechnology Center, Purdue University, West Lafayette, IN USA; 3 Center for Implantable Devices, Purdue University, West Lafayette, IN USA; 4 Department of Psychological Sciences, Purdue University, West Lafayette, IN USA; 5 Purdue Institute of Integrative Neuroscience, Purdue University, West Lafayette, IN USA; 6 Indiana University School of Medicine, Indianapolis, IN USA

**Keywords:** Electrical and electronic engineering, Structural properties

## Abstract

The vagus nerve (VN) plays an important role in regulating physiological conditions in the gastrointestinal (GI) tract by communicating via the parasympathetic pathway to the enteric nervous system (ENS). However, the lack of knowledge in the neurophysiology of the VN and GI tract limits the development of advanced treatments for autonomic dysfunctions related to the VN. To better understand the complicated underlying mechanisms of the VN-GI tract neurophysiology, it is necessary to use an advanced device enabled by microfabrication technologies. Among several candidates including intraneural probe array and extraneural cuff electrodes, microchannel electrode array devices can be used to interface with smaller numbers of nerve fibers by securing them in the separate channel structures. Previous microchannel electrode array devices to interface teased nerve structures are relatively bulky with thickness around 200 µm. The thick design can potentially harm the delicate tissue structures, including the nerve itself. In this paper, we present a flexible thin film based microchannel electrode array device (thickness: 11.5 µm) that can interface with one of the subdiaphragmatic nerve branches of the VN in a rat. We demonstrated recording evoked compound action potentials (ECAP) from a transected nerve ending that has multiple nerve fibers. Moreover, our analysis confirmed that the signals are from C-fibers that are critical in regulating autonomic neurophysiology in the GI tract.

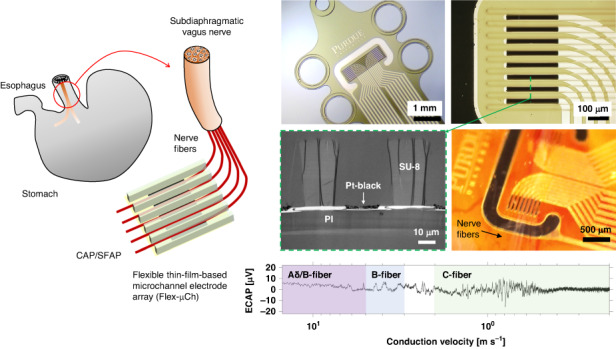

## Introduction

The vagus nerve (VN) is the tenth cranial nerve pair that primarily mediates parasympathetic functions of the body via connections from the medulla oblongata to various organs in the body, including heart, lungs, liver, spleen, and gastrointestinal (GI) tract. As a part of autonomic nervous system, the VN plays a critical role in regulating a wide range of bodily functions, including heart rate, respiration, immune response, and digestion. Knowledge of the structure-function relationships of the left/right vagal nerves, along with their typical signaling patterns, are substantially incomplete, which continues to limit the introduction of more sophisticated technologies to both diagnose or treat autonomic dysfunctions in tissues supplied by the VN.

The GI tract, a critical part of the digestive system, is regulated by enteric nervous system (ENS). The ENS contains 400–600 million neurons and is sometimes called the “second brain” due to its complexity and ability to operate independently from the central nervous system (CNS)^1^. The VN connects the CNS and ENS to regulate physiological activities in the GI tract^2^. Meanwhile, alteration of VN activities (e.g., as a result of injury, disease, or genetic mutation) can disrupt the physiology of the GI tract.

The VBLOC Therapy Maestro system is an FDA-approved device intended to restate the hunger-suppressing effect of subdiaphragmatic vagotomy^3,4^. Although trial studies report treatment group of study participants experienced significant weight loss compared to sham group^3^ and sustained the excessive weight loss compared to control group for 24 months^4^, the efficacy of the treatment is known to be modest^4,5^. Moreover, several animal studies reported that excitation – not the blocking – of VN activity by the VBLOC system resulted in reduced food intake and weight loss^6–8^.

The incomplete knowledge about the efficacy of the therapeutic intervention of the VN might be attributed, in part, to the lack of information regarding the structure-function relationships of the VN and ENS of GI tract. While there are studies that show dysfunction of the VN is related to a variety of health conditions such as inflammatory bowel disease (IBD)^9^, obesity^10^ and gastroparesis^11^, the underlying mechanisms are still not well understood^12^.Optimal bioelectronic treatment methods that utilize the VN will require a more complete understanding of the neurophysiology of VN, ENS, as well as other physiological systems.

Ambulatory recording of naturalistic patterns of single-fiber action potentials (SFAPs) could provide a rational basis for understanding the structure-function relationships of the VN and its effectors, and their roles in health and disease. With the development of our knowledge about the distinct patterns of neural signals from individual nerve fibers in the branches of the subdiaphragmatic VN, it is likely that the underlying mechanisms of GI-tract dysfunctions associated with VN will become more precisely defined.

To study VN neurophysiology, researchers typically use two types of neural interface devices: (1) Intraneural probe electrode array type^13–17^; (2) Extraneural cuff electrode type^18–21^:

Jiman et al. developed a carbon fiber based intraneural microelectrode array, which have shown the capability to record clusters of different spikes that also included conduction velocity estimates^14^. Although they were able to record propagating activities with slow conduction velocity (0.7 m s^−1^), which are associated with unmyelinated C-fibers, they discussed that only some of the recording sites were able to capture propagating activities, possibly due to the intrinsically incomplete alignment of the nerve fibers to the electrode array for the intraneural probe type devices^14^.

Cuff electrodes can also record signals from VN via extraneural electrodes^18–21^. Zanos et al. used cuff electrodes to study about decoding cytokine-specific sensory neural signals in the VN of mice, showing that these signals could be discriminated based on their firing rates^20^. However, there were no direct reports on the conduction velocity of the signals, and the signals are regarded as compound action potentials (CAP) since the cuff electrode wraps the entire bundle of nerve fibers.

We seek a VN recording solution that limits the potential damage to the nerve/nerve fibers that may result from penetrating microelectrodes, while allowing for the discrimination of single-fiber action potentials (SFAPs) and their conduction properties. Among the candidates, microchannel electrode arrays may provide an ideal middle-ground solution to enable safer, more advanced neurophysiologic studies of the VN^22–29^. Microchannel electrode devices normally feature channel structures with micrometer scale dimensions (50–100 µm of channel width and height) and array(s) of electrodes embedded in each channel to interface with a finite bundle of nerve fibers that are separately placed in each channel^22–28^. Typically, microchannel electrode arrays require regeneration of nerve fibers: For in-vivo studies, nerve tissues are transected and microchannel structures are placed as conduits for the regeneration of nerve fibers^22,25,26^, while for in-vitro studies, neurites are grown into the microchannels to record signals^27,28^.

However, there are several studies that have used nerve teasing methods to separate a nerve bundle into smaller bundles of nerve fibers before placing them into a microchannel electrode system, which does not require regeneration of nerve fibers^23,24,29^. Chew et al. demonstrated a closed-loop neuroprosthetic system that uses a polydimethylsiloxane (PDMS) based microchannel device (200 µm thick), which was implanted in teased dorsal roots (dorsal rootlets) within the rat vertebral column to measure bladder fullness and to prevent spontaneous voiding episodes in rats with neurogenic bladder caused by spinal cord injury^24^.

The concept of a microchannel device with teased fibers can be a promising approach for investigating the fiber/organ-specific neurophysiology of the subdiaphragmatic VN and the GI tract. However, to achieve this potential, the design of the device must be optimized. Specifically, the volumetric footprint of the device needs to be minimized to reduce the mechanical stress on the nerve and surrounding structures, which have complex and dynamic shapes. At the same time, the device must maintain high flexibility to ensure accurate and reliable measurements. By addressing these design challenges, microchannel devices with teased fibers hold significant potential for advancing our understanding of the subdiaphragmatic VN and its role in GI function.

**Fig. 1:**
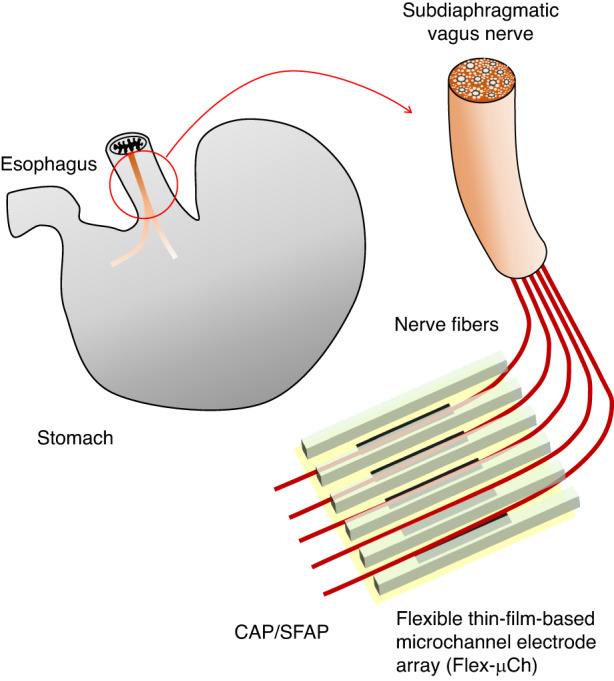
Schematic illustration of the concept of using the flexible thin film based microchannel electrode array device to interface subdiaphragmatic VN to investigate neurophysiology of GI tract

In this paper we present a novel flexible microchannel electrode array (Flex-µCh) that can interface with the subdiaphragmatic VN in rats (Fig. 1), specifically the ventral gastric branch of the VN (VGVN). We designed, fabricated, and characterized the device and demonstrated its functionality via surgical implantation to the VGVN and recording of neural signals from the VGVN (*N* = 7). We successfully recorded electrically evoked compound action potentials (ECAP) with different conduction velocities from the multiple channels of the device.

## Design and fabrication

### Design of the microchannel electrode array device

**Fig. 2:**
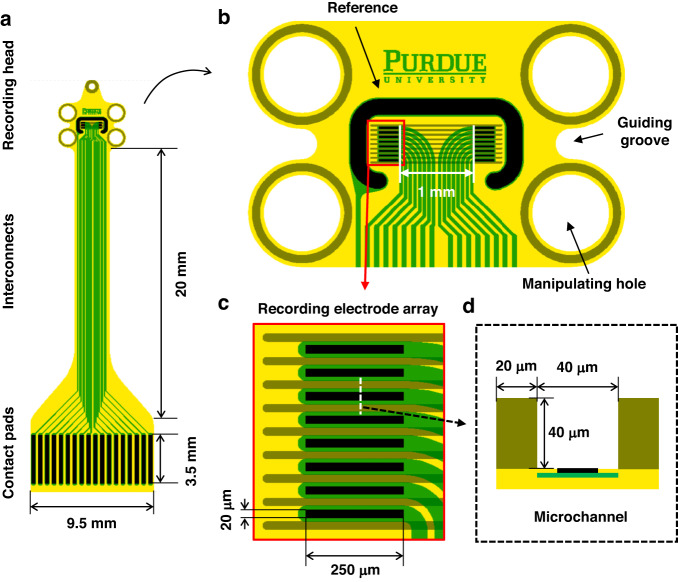
Design of the flexible thin film based microchannel electrode device. **a** Three compartments (contact pads, interconnects, and recording head) of the device. **b** Layout of the recording head of the devices with characteristic features. **c** Zoomed in layout of the recording electrode array within the microchannel with the dimension of the electrode (width: 250 µm, height: 20 µm). **d** Cross-sectional view of the microchannel and the electrode with the dimensions of the channel structure (channel spacing: 40 µm, channel wall height: 20 µm, channel wall depth: 40 µm)

The Flex-µCh device consists of three key components, namely contact pads, interconnects, and recording head (Fig. 2a). We designed the contact pads to be compatible with commercially available Zero Insertion Force (ZIF) connector, which is widely used for industrial electronic devices: The pitch of the contact pads is 0.50 mm with the width and height per pad of 0.25 mm and 3.5 mm respectively. The total width of the contact pads including the substrate is 9.5 mm, which is based on the standard for ZIF connector: (number of the contacts + 1) × 0.5 mm, where the number of the contacts for our device is 18. The length of the interconnect parts is 20 mm, which is enough to ensure facile implantation of the electrodes part to the VGVN without the contact pads part being too close to the implantation site.

The recording electrodes within each microchannel are separated 1 mm apart (edge-to-edge) to give enough conduction distance between them to separate latencies of the slowly conducting C-fiber signals (conduction velocity <2 m s^−1^) to a resolvable degree^30^. A large reference electrode surrounds the microchannel/electrode array (GSA = 838100 µm^2^) to effectively reduce the noise coming from the outside of the recording sites with the closest distance from the edge of the reference electrode to the edges of the microelectrodes kept at 170 µm (Fig. 2b). The design of the recording head features four manipulating holes (inner diameter = 1 mm) at the corners and two guiding grooves at the sides (Fig. 2b): The manipulating holes are mechanically reinforced with SU-8 to prevent folding while manipulating with forceps or other surgical instruments. The guiding grooves are designed to facilitate the alignment of the nerve along the microchannels during the implantation process.

The recording electrode array at each side (left hand side or right hand side) of the recording head as well as the microchannel walls has 60 µm of pitch with height and width of the electrode as 20 µm and 250 µm respectively (GSA = 5000 µm^2^) (Fig. 2c). The cross section of the microchannel features 40 µm of spacing; 20 µm of height; 40 µm of the depth (Fig. 2d). We determined the dimensions of the microchannel based on the diameter of rat VGVN (100 µm)^31^, which will be teased to several fibers with smaller diameters.

### Microchannel electrode array device fabrication

**Fig. 3:**
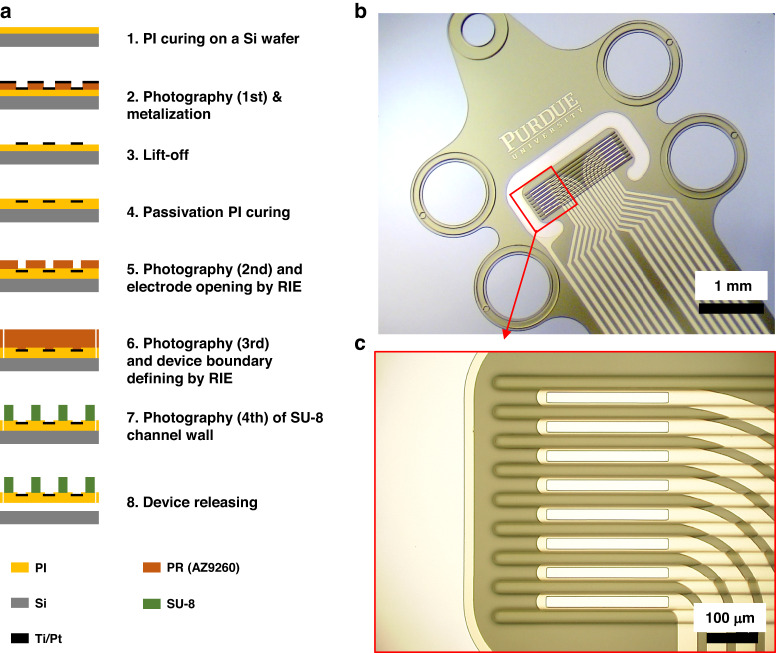
Fabrication of the device. **a** Cross-section flow diagram of the device fabrication process. **b** A photographic image of the device as fabricated on the silicon wafer. **c** A microscopic image of the recording electrode array within the microchannel structure

The Flex-µCh devices were fabricated using well-established cleanroom microfabrication processes (Fig. 3a). PI resin (PI 2525, HD MicroSystems, Parlin, NJ, USA) was spin-coated (thickness = 10 µm) on a 4-inch silicon (Si) wafer and cured at 300 °C for 1 h. The PI substrate was cleaned with toluene, acetone, isopropyl alcohol, and deionized water (DI water) before all photolithography processes. A positive photoresist (PR, AZ9260, MicroChem, Newton, MA, USA) was spin-coated and soft-baked at 110 °C. A maskless aligner (MLA150, Heidelberg Instruments, Germany) was used to directly write the metal trace pattern with UV laser (405 nm). After the exposure, the wafer was developed by using a developer (AZ 400 K, MicroChem, Newton, MA, USA) with 1:3 dilution (1 part of AZ 400 K, 3 parts of DI water). After the first photolithography, the wafer was treated with oxygen plasma (100 W, O_2_ 50 sccm) for 2 min to descum and clean the surface of PI. The wafer was sputtered with 10 nm of titanium (Ti) and 100 nm of platinum (Pt) followed by lift-off. The wafer was spin-coated with another layer of PI (PI 2545, HD MicroSystems, Parlin, NJ, USA) with 1.5 µm of thickness for the passivation and cured at 300 °C for 1 h. The wafer was spin-coated with PR for the second photolithography to define electrodes and contact pads. After the exposure and developing process, the contact pads and the electrodes were exposed by using reactive ion etch (RIE) with 50 sccm of O_2_ at 100 W in 50 mTorr for 10 min. After the RIE process, the PR layer was removed by acetone and spin-coated again with PR for the third photolithography followed by RIE process to define the boundary of the devices. After removing the remaining PR with acetone, the wafer was spin-coated with SU-8 resin (SU-8 2050, Kayaku Advanced Materials, Newton, MA, USA) for the fourth photolithography to define the walls of the microchannels. After developing process, the SU-8 layer was hard-baked at 200 °C for 30 min to enhance mechanical stability. Figure 3b shows the image of fabricated device on a silicon wafer before the releasing process. The devices were released from the Si substrate by etching the native oxide layer by using buffered oxide etch (BOE) solution. The released devices were rinsed with DI water 5 times before further usage.

**Fig. 4:**
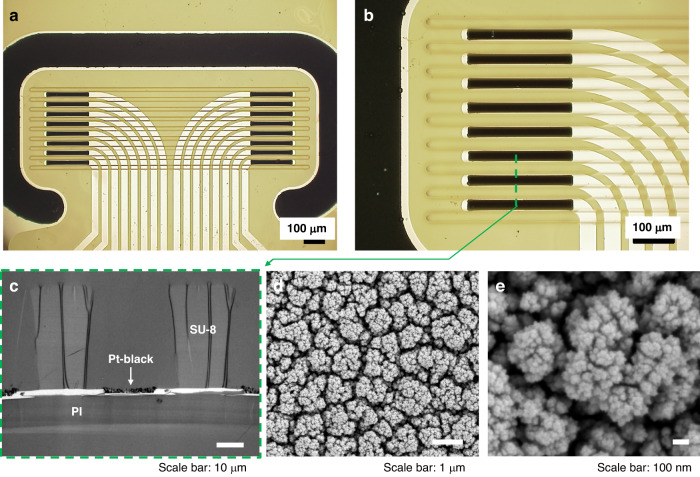
Pt-black coating of the recording electrodes and the reference electrode. **a** A microscopic image of the recording head after the Pt-black coating. **b** Recording electrode array in a zoomed in microscopic image. **c** TEM image of the cross-section of the microchannel electrode device. SEM images of the surface of the Pt-black coating with self-similar morphological features in micrometer scale (**d**) and nanometer scale (**e**)

The recording electrodes are defined in between the SU-8-based microchannel walls (Fig. 3c). The surface of Pt was platinized by electroplating to form a highly rough Pt-black layer to improve the impedance^32^. The plating solution was prepared by mixing 17.5 mM of hexachloroplatinic acid (262587, Sigma-Aldrich, St. Louis, MO, USA) and 0.03 mM of lead (II) acetate trihydrate solution (316512, Sigma-Aldrich, St. Louis, MO, USA). Pt-black was coated on the surface of the electrodes by applying the electric potential that negates the open circuit potential of the working electrode versus Ag/AgCl reference electrode in the plating solution. The Pt-black coating was controlled by the total injected charge density (4.7 C cm^−2^) that doesn’t cause overgrowth of the grain at the edge of the electrode. The electrodes in between the microchannels are coated with the Pt-black within their geometric boundaries (Fig. 4a, b). The dendrites of the Pt-black coating extend to the surface of the encapsulation PI layer as shown in the cross-sectional TEM image (Fig. 4c). The Pt-black coating has cauliflower-like fractal nano morphology with self-similar dendritic grains from micrometer scales (Fig. 4d) to nanometer scales (Fig. 4e)^32^.

### Electrochemical characterizations of the electrodes

Electrochemical interface of the electrodes of the Flex-µCh device was characterized by conducting cyclic voltammetry (CV) and electrochemical impedance spectroscopy (EIS). CV was conducted by using a multichannel potentiostat (MultiPalmSens4, PalmSens, Houten, Netherlands) in PBS solution (ThermoFisher Scientific, Waltham, MA, USA). Three-electrode configuration was used: A microelectrode on the Flex-µCh device as a working electrode, a graphite rod electrode with 5 mm of diameter as a counter electrode, and Ag/AgCl with 3 M KCl reference electrode (RE-1CP, ALS Co. Ltd., Tokyo, Japan). The potential range of the CV was set from −0.6 V to 0.8 V versus Ag/AgCl reference electrode with 50 mV s^−1^ of scan rate for 25 cycles, where CV curves stabilize. EIS was conducted using a potentiostat (SP-200, Bio-Logic Inc, Seyssinet-Pariset, France) with the same electrode configuration in the same PBS solution immediately after the CV was conducted. Cathodic charge storage capacity (CSC_c_) was calculated from the charge storage capacity (CSC) using the following equation^33,34^:

1 $${CSC}=\frac{1}{\nu A}{\int }_{{\!E}_{c}}^{{E}_{a}}\left|i\right|{dE}$$

Where *E* is the potential versus Ag/AgCl reference electrode; *i* is the current; *E*_*a*_ and *E*_*c*_ are positive and negative vertices for CV respectively; *A* is the geometric surface area of the electrode; *ν* is the scan rate.

## In-vivo demonstration of microchannel device functionality in rat

### Animal experiment

All rodent experiment procedures were approved by Purdue Animal Care and Use Committee (PACUC) under the protocol number 1112000390. Seven male Sprague-Dawley rats (Envigo, male, 250–450 g) were used for in-vivo testing. Animals were housed in a 12-h light/dark cycle at a constant humidity level and temperature. Animals were anesthetized by isoflurane (5.0% for the induction, 0.5–2.5% for the maintenance) with O_2_ gas (flow rate: 0.3–0.5 L/min) using a ventilator (Vetamac VAD Compact Tabletop Anesthesia Machine, Vetamac, Rossville, IN, USA). Core temperature was maintained at 35.9–37.5 °C by a heat pad. After induction of anesthesia, animals were moved to a surgical frame in a supine position. For analgesia, 0.04 mL of butorphanol tartrate (0.5–2 mg/kg) was injected subcutaneously through the dorsal skin every 4 h. Ten minutes after the injection of analgesia, the surgical site was shaved by using a clipper and cleaned by scrubbing with betadine swab sticks and DI water three times. Saline was injected subcutaneously (1–4 mL per hour) to prevent dehydration of the animals.

### Left cervical vagus nerve isolation and cuff implantation

For each animal experiment (total *N* = 7), we implanted a cuff electrode to the left cervical vagus nerve (LCVN) to stimulate the nerve and record the ECAP from our microchannel electrode array device. After shaving and cleaning the surgical site with a betadine solution, a midline incision was made from the mid-point of the caudal ends of mandibles to the manubrium of sternum. Blunt dissection was performed until the sternohyoid, omohyoid, and sternocleidomastoid muscles are visible. The connective tissue between the left sternocleidomastoid and the omohyoid/sternohyoid was dissected carefully to locate the carotid sheath. A retractor was used to hold the sternocleidomastoid and the sternohyoid apart. The left lateral distal part of the omohyoid was carefully separated to open a triangular window to get access to the carotid sheath, which contains LCVN along with carotid artery and jugular vein. The LCVN was isolated from the carotid sheath by carefully performing blunt dissection parallel to the axial direction of the nerve using iris scissors until the nerve was clearly visible. A cuff electrode with 400 µm of inner diameter (FNC-400-V-R-2C-30, MicroLeads, Somerville, MA, USA) was implanted to the isolated LCVN for neural stimulation.

### Subdiaphragmatic vagus nerve isolation and device implantation

**Fig. 5:**
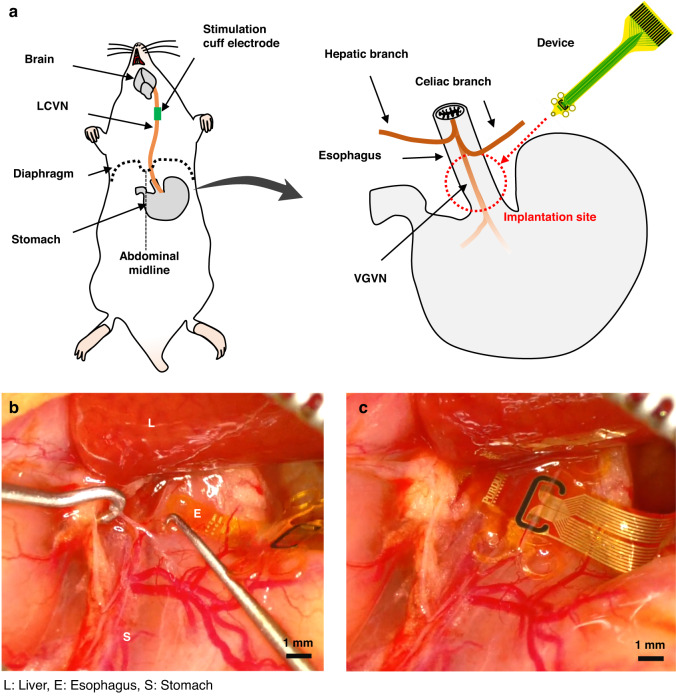
Surgical implantation of the device to the VGVN. **a** Schematic illustration of the relevant anatomical structures and the surgical implantation of the device to the VGVN. **b** A surgical microscopic image of the VGVN isolation procedure using a fine surgical hook at the implantation site with multiple surrounding tissue structures (L Liver, E Esophagus, S Stomach). **c** A surgical microscopic image of the implantation site after the device implantation to the VGVN. A translucent structure placed on the recording head is the isolated VGVN

The target implantation site is located on the ventral surface of the esophagus caudal to the hepatic and celiac branches of the subdiaphragmatic vagus nerve (Fig. 5a). After the cuff electrode implantation to the LCVN for stimulation of nerve activity, abdominal surgery was performed to get access to the VGVN Flex-µCh device implantation site. After shaving hair and cleaning the abdominal surgical site with betadine solution, a midline incision was done starting from the middle of the abdomen of the rat (Fig. 5a). After careful initial incision of the skin layer using a surgical scalpel, the muscle layer was carefully dissected by using scissors following the midline until xiphoid cartilage is visible. The abdominal surgical site was carefully retracted by using magnetic fixator-based retractors. The hepatogastric ligament and hepatoduodenal ligament were dissected to separate the liver and the stomach. The liver was carefully retracted rostral so that the esophagus is visible. The VGVN was carefully isolated from the surface of the esophagus by using a surgical micro point (10065-15, Fine Science Tools, GmbH, Germany). The Flex-µCh device was inserted between the VGVN and the esophagus while the isolated VGVN was lifted up with a nerve hook (Fig. 5b). Due to the concave guiding grooves at the side of the device, the VGVN was self-aligned on the microchannels (Fig. 5c). After the device implantation, conduction distance for the ECAP was measured between the distal end of the cuff electrode and the proximal end of the recording electrodes on the Flex-µCh device.

### Neural stimulation and recording

Custom MATLAB software developed by Ward et al. was used to control the timing and parameters for LCVN stimulation^35^ with the cuff electrode implanted to the LCVN. The stimulation was performed via cathode-first, alternating monophasic stimulation^32,35^: An initial cathodic monophasic pulse to stimulate the nerve and a subsequent anodic monophasic pulse for charge balancing. The stimulation was fed through a battery-powered stimulus isolator (BSI-1A, BAK Electronics, Inc, Umatilla, FL, USA), which converted the time-varying voltage waveform into a constant current equivalent. Pulse current amplitudes were set at different values (0, 0.05, 0.1, 0.2, 0.4, 0.6, and 0.8 mA); pulse duration was set to 0.6 ms to activate unmyelinated C-fibers of the vagus nerve; and pulse repetition frequency was set to 1 Hz to ensure capturing signals from C-fibers which have conduction velocity slower than 2 m s^−1^.

A multichannel amplifier headstage (RHD 16-channel C3334, INTAN technologies, Los Angeles, CA, USA) was used for the data acquisition in the animal experiment. The sampling rate for the recorded signal was set at 25 kHz, and the bandwidth was set at 0.98 Hz–7.60 kHz. Digital highpass filter was applied to the signal with cut-off frequency at 10 Hz to effectively reduce respiration and cardiac artifacts. A polyetheretherketone (PEEK) film (Cat. No. 4671T61, McMaster-Carr, Elmhurst, IL, USA) was attached to the back side of the contact pads of the device for a stiff backing as introduced in a previous study^36^ for connection to a ZIF connector (FH12-18S-0.5SH, Hirose Electric Group, Kanagawa, Japan), which was soldered on a custom PCB to adapt to an Omnetics connector (A79040-001, Omnetics Connector Corporation, Minneapolis, MN, USA) for the connection to the amplifier headstage (Supplementary Fig. S1).

## Results

### CV and EIS of Pt-black coated electrodes

**Fig. 6:**
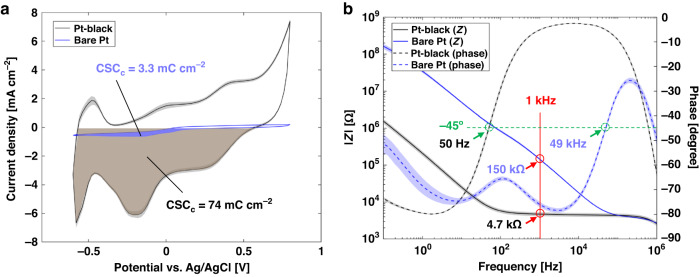
Electrochemical characterizations of the recording electrodes. **a** CV plot of the Bare Pt electrodes (blue) and the Pt-black coated electrodes (black). Solid lines: Mean CV plots (*N* = 8 each). Shaded region along the solid lines: 95% CI (*N* = 8 each). **b** EIS Bode plot of the Bare Pt electrodes (blue) and the Pt-black electrodes (black). Solid lines (blue and black): Mean magnitude of impedance (|Z|) with respect to frequency (*N* = 8 each). Dashed lines (blue and black): Mean phase delay of the electrical response (*N* = 8 each). Intercepts of solid lines to the vertical red solid line indicate |Z| at 1 kHz for each electrode. Intercepts of dashed lines to the horizontal green dashed line indicate cut-off frequency at −45° of phase delay. Shaded region: 95% CI (*N* = 8 each)

The mean CV curve (*N* = 8) at 25^th^ cycle from the Pt-black (“Pt-black”) electrodes show significant enlargement compared to the curve from bare Pt (“Bare Pt”) electrodes (Fig. 6a): The CV curve of Pt-black also shows all the features of the Pt/PBS electrochemical interface such as peaks from oxide reduction, hydrogen adsorption, hydrogen desorption, and surface oxidation^37^. With substantially enlarged electrochemically active surface of the Pt-black, the CSC_c_ of the Pt-black electrodes (73.8 ± 6.6 mC cm^−2^) is 22 times higher than the CSC_c_ of the Bare Pt electrodes (3.30 ± 0.11 mC cm^−2^).

The EIS Bode plot of Pt-black and Bare Pt electrodes (*N* = *8* each) show significant enhancement of impedance response for neural recording represented by the cutoff frequency and impedance at 1 kHz (Fig. 6b). The cutoff frequency (f_cutoff_) is defined as the frequency where the phase angle is −45° and at this frequency, the magnitude of electrochemical impedance (|Z|) transitions from the predominantly resistive domain, where |Z| is relatively lower and less sensitive to frequency, to capacitive domain, where |Z| starts to increase exponentially with respect to frequency^37^. Pt-black electrodes show three orders of magnitude lower f_cutoff_ (50 Hz) compared to Bare Pt (49 kHz), which covers wider frequency range without severe distortion of signals. Also, the values of |Z| at 1 kHz, which is more simplified but widely used value to evaluate recording performance, show that Pt-black electrodes (4.75 ± 0.76 kΩ) are 31 times lower than Bare Pt electrodes (146 ± 9.7 kΩ).

### ECAP signal measurement using the microchannel electrode array device

**Fig. 7:**
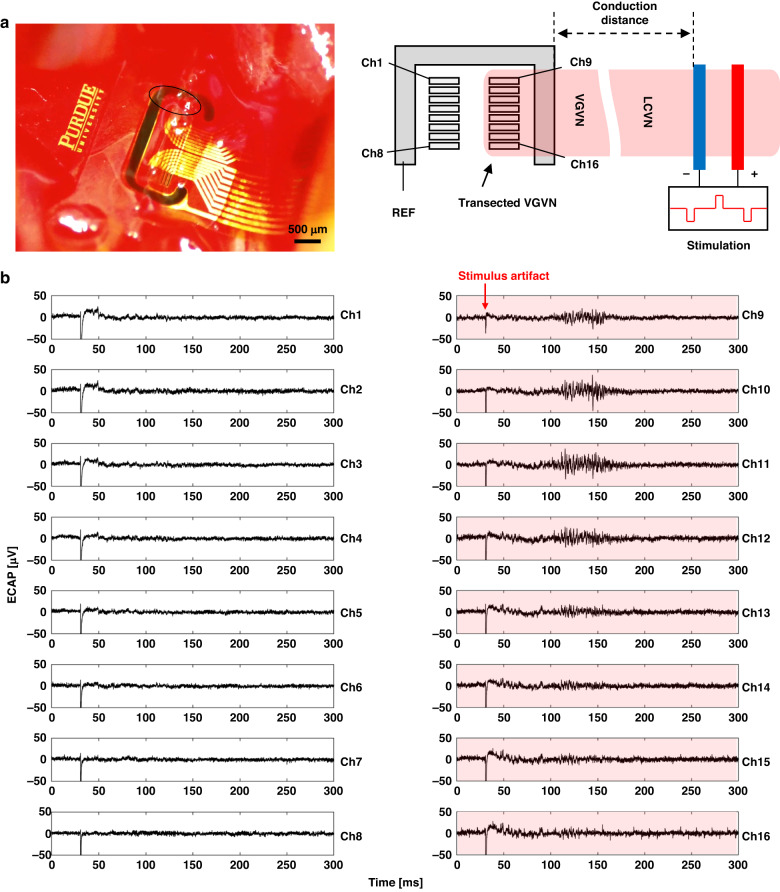
Acute demonstration of simultaneous recording of ECAP signals from the electrode array within the microchannel. **a** Surgical microscopic image (left) and the schematic illustration of the stimulation and recording configuration. Transected nerve ending was placed on the righthand side of the electrodes (Ch9-Ch16, indicated by the solid black circle in the surgical microscopic image). **b** ECAP signals from all the channels. Red shaded plots indicate the channels with the transected VGVN placed on (Ch9-Ch16)

To demonstrate the neural signal recording capability of the microchannel electrode array, we transected the VGVN, and placed the proximal end on the electrodes at the right-hand side of the device (Ch9-Ch16, Fig. 7a). The data acquisition began 2–4 h after the initial incision and completed within 1–2 h. Before the stimulation, 10 s of signals were recorded to gauge the noise floor: The root mean square of noise floor (RMS_noise_) for the signals from all the channel was 2.80 ± 0.15 µV. ECAP signals are acquired from the electrode array at cathodic stimulation phase (Fig. 7b). After the stimulus artifact from the cathodic stimulation (pulse current amplitude = 0.6 mA; pulse duration = 0.6 ms), dispersed volleys of ECAP appeared at the Ch9-Ch16 electrodes where the transected nerve ending was placed, whereas there were no clearly observable responses from the other electrodes (Ch1-Ch8) without the nerve placed on. ECAP signals at different stimulus pulse currents (0.05, 0.1, and 0.6 mA) show recruitment of different fiber groups at different conduction velocity ranges (Supplementary Fig. S2a–c).

To compare the ECAP signals with multiple peaks, we analyzed maximum peak-to-peak amplitude (Max V_pp_) of all the cathodic phases within a limited latency range where peaks appear (5–250 ms): Limiting the latency range for analysis can effectively remove the stimulus artifacts from the data to avoid misclassifying stimulus artifact as physiologic data. Max V_pp_ was obtained by applying “peak2peak” function of MATLAB within the 2 ms of moving time frame throughout the limited latency range (5–250 ms). The 2 ms timeframe was determined to effectively capture the peak-to-peak amplitude of the individual diphasic volley that has one negative peak followed by one positive peak.

**Fig. 8:**
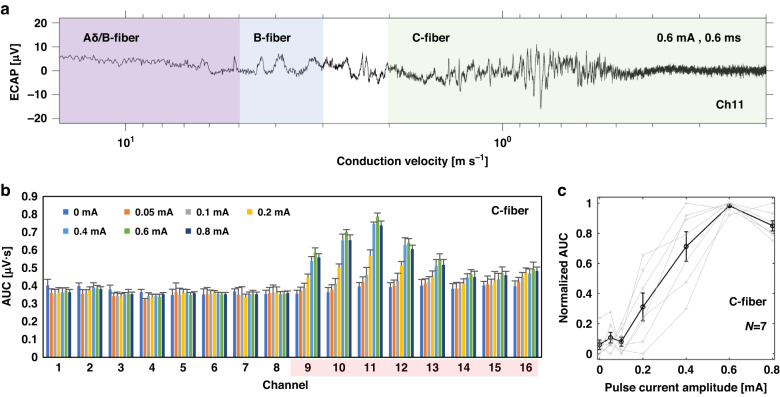
Analysis on the conduction velocity of the ECAP volleys from the acute demonstration. **a** ECAP plot with respect to conduction velocity estimated from the measured conduction distance (pulse current amplitude: 0.6 mA, pulse duration: 0.6 ms). **b** Area under the curve (AUC) for the ECAP volleys at the C-fiber conduction velocity range for all the channels (pulse duration: 0.6 ms). Red-shaded channels (Ch9-Ch16) indicate the electrodes with VGVN nerve ending placed on. **c** Normalized AUC profile with respect to pulse current amplitude in multiple animals (pulse duration: 0.6 ms, *N* = 7 animals) from the channels that showed highest magnitude for the ECAP in C-fiber conduction velocity range. Gray: individual experiments; black: mean of all experiments; error bars: standard error of the mean

ECAP volleys became visually evident at different latencies in response to 0.1 mA stimulation (Supplementary Figure S2b), with max V_pp_ of 37.6 µV compared to 0.05 mA stimulation (Supplementary Fig. S2a) with max V_pp_ of 23.2 µV, and the response becomes stronger at 0.6 mA (Supplementary Fig. S2c) especially between 70–170 ms of latency range with max V_pp_ of 72.8 µV. With 73 mm of measured conduction distance, the latency range corresponds to the C-fiber conduction velocity range (<2.0 ms^−1^)^38^. Activities from other fiber groups, such as Aδ and B-fiber are also present, but the C-fiber activities were recorded only in response to stimulation with higher intensity (Fig. 8a); therefore, we focused on C-fibers for further analysis.

We determined the signal-to-noise ratio (SNR) of the ECAP signal from the result in Fig. 8a using following equation^29^:

2 $${SNR}={10\log }_{10}{\left(\frac{{{Max}V}_{{pp}}}{{{RMS}}_{{noise}}}\right)}^{2}$$

Based on the RMS_noise_ (2.92 µV) and the Max V_pp_ (72.8 µV) values, the calculated SNR of ECAP signal is 28.0 dB.

We calculated area under the curve (AUC) for the ECAP signals at the conduction velocity range for unmyelinated C-fibers. The results show that the AUCs from Ch9-Ch16 electrodes show similar trends of nerve fiber recruitment (Fig. 8b). We repeated the ECAP recording experiments in multiple animals (*N* = 7) to obtain the recruitment profile of the C-fiber activities in VGVN. In each experiment, we have identified the channel that showed the highest magnitude of the AUC at the pulse current amplitude that showed strongest ECAP response at the C-fiber conduction velocity range. We normalized the AUC per each experiment to mitigate confounding factors such as animal variability and differences in the nerve fiber-electrode contact by using following equation:

3 $${Normalized\; AUC}(i)=\frac{{AUC}(i)-{{AUC}}_{\min }}{{{AUC}}_{\max }-{{AUC}}_{\min }}$$

Where $i$ is the pulse current amplitude, ${{AUC}}_{\max }$ and ${{AUC}}_{\min }$ are the maximum and minimum AUCs respectively. The normalized AUC increased until 0.6 mA of pulse current amplitude and slightly decreased at 0.8 mA (Fig. 8c).

In one of the experiments, in response to 0.6 mA of pulse current, where the AUC was greatest, we observed a progressive delay in C-fiber response latency with each repetition of the stimulus (pulse repetition frequency = 1 Hz) throughout the 10 s of train duration (Supplementary Fig. S3a). The latency delay became more notable for slower fiber types with a tendency to recover initial latencies following a 7 s pause between trials (Supplementary Fig. S3b). This latter result suggests a characteristic feature of stimulus induced depression of neuronal excitability (SIDNE)^39^.

## Discussion

The Flex-µCh device has smaller volumetric footprint (11.5 µm thick) compared to other microchannel devices for teased fibers that use PDMS substrate (200 µm thick). In this report, we demonstrated successful implantation of the Flex-µCh device to the rat VGVN, which is under constant mechanical motion from respiration and organ motility. The electrodes interfacing with the transected VGVN endings (Ch9-Ch16) recorded clear ECAP responses, whereas the other electrodes (Ch1-Ch8) that did not interface with the VGVN did not record signals (Fig. 7b), which shows the potential of un-coupled recording of individual activities from teased nerve fibers.

Our result clearly captured highly dispersed ECAP volleys in the C-fiber conduction velocity range (Fig. 8a), which were evoked from LCVN and traveled down to VGVN: The result confirm previously reported experimental and modeled ECAP signals in rat, where LCVN was stimulated and ECAP was recorded from the ventral branch of subdiaphragmatic vagus nerve^40^. The result also confirmed that C fibers are readily excitable at the level of the cervical VN with relatively low activation thresholds (and measurable downstream at the level of the VGVN), and that a subset of C fibers can be excited at the level of the cervical vagus nerve before all A and B fibers have been recruited. Unmyelinated C-fibers, which comprise over 99% of the VGVN^41^, play a critical role in regulating various aspects of GI function, including motility, secretion, and inflammation in the GI tract. Our Flex-µCh device is capable of recording the activity of unmyelinated C fibers.

In one of our experiments, we obtained ECAP signal with 28.0 dB of SNR, which can be regarded as a high-quality signal based on a previous study^29^: Gribi et al. evaluated an algorithm that can calculate the velocity of an electrically evoked neural signal, which is recorded from a teased dorsal rootlet of rat that has similar diameter as VGVN (~100 µm) in a microchannel-based ex-vivo setting, by comparing the result of calculated velocity of the signal with a reference value and identifying the success rate, which is defined as the ratio of successful calculation within 10 repetitions of a neural signal. From the evaluation result, they suggested a linear model about the relationship between success rate, velocity of the signal and SNR. According to the model, the algorithm would achieve success rate higher than 0.8 (out of 1) for the signals with 20–30 dB of the SNR, which implicates that our result could be regarded as a high-quality signal given that they used results from the signals with 0.8–1 of success rate to show the highest performance of their platform^29^.

While we have demonstrated recording capability of C-fibers using our Flex-µCh device in the VGVN of rat, we acknowledge several limitations to the current study. First, a better nerve teasing method should be established to fully make use of microchannel electrode array (we resorted to recording from the transected end of the VGVN in this report). Chew et al. used a pair of fine glass pulled probe to tease L6 dorsal root of rat into five to nine rootlets with around 100 µm diameter to place them into microchannels with 100 µm spacing without a transection^24^. However, the method has not been used further to tease a nerve with 100 µm or smaller diameter in an in-vivo setting. Rat VGVN is known as a single fascicular nerve with 100 µm of diameter and teasing it without transection might require a specialized tool that has ultra-fine feature to dissect the nerve without causing damage to the nerve fibers and blood vessels in the nerve.

**Fig. 9:**
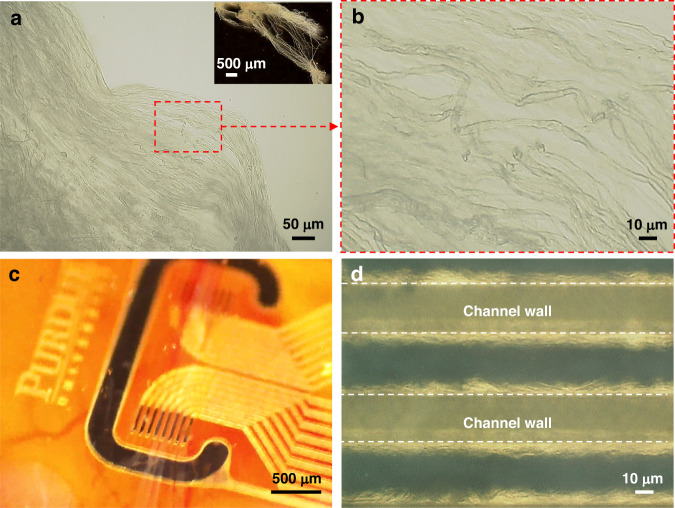
Optical microscope images of nerve fibers from the LCVN and the scene of the nerve fibers-device interface. **a** Separated bundles/individual strands of nerve fibers from the LCVN (inset: the LCVN teased to smaller structures). **b** Nerve fibers observed with a higher magnification (×1000). **c** Flex-µCh device interfacing with the teased VGVN. **d** Nerve fibers from harvested VGVN placed within the microchannel structure

Rat VGVN is composed mostly of unmyelinated C-fibers (99.6%, 7445 ± 314 fibers), with 0.77 ± 0.02 µm of average diameter, and a handful number of myelinated fibers (0.4%, 31 ± 3 fibers) with 2.08 ± 0.06 µm of average diameter^41^. Under the benchtop optical microscope, the Remak bundles of unmyelinated C-fibers or individual myelinated fibers might be observable at high magnification (Fig. 9a, b, see “Supplementary experimental details” in the supplementary information for the experimental details). However, under the surgical microscope in the surgery, the teased chunk of VGVN might be observable (Fig. 9c), but it is hard to stay focused at a high magnification to resolve the individual fibers due to the limit of lens performance and the motion from respiration and organ motility. Figure 9d shows the scene of nerve fibers placed in the microchannels of the Flex-µCh device observed with a benchtop microscope. With an improved experimental setting, such as high-quality microscope for the surgery and a mechanism to isolate the tissue from the motion, it might be possible to better control the placing of teased nerve fibers into the microchannels.

In addition to the difficulties in the teasing method, stable detection of natural patterns of spontaneously evoked SFAP from the VGVN, which is in a highly dynamic environment, also remains a challenge and ongoing goal of our efforts. Although several in-vitro studies report extracellular SFAP recordings^29,42^, the studies are limited to electrically evoked activities in a well-controlled environment. VGVN is surrounded by tissues that generate mechanical motions including diaphragm (source of respiration), esophagus (esophageal motility), and stomach (gastric motility). Although our Flex-µCh device can mitigate the mechanical interaction due to its flexibility and minimal volumetric footprint, the artifacts from the mechanical motion could add more challenges to the signal processing for SFAP detection: For example, the amplitude of the single unit spikes from the cervical vagus nerve can be modulated by the respiration, and therefore it requires advanced signal processing methods to properly detect the signal of interest^20^. With the presence of multiple mechanical artifacts that might modulate the SFAP from the VGVN in the same manner as in the cervical level, further advanced signal processing might be necessary to properly detect the SFAP from the VGVN.

Musick et al. reported an ambulatory chronic recording of single spike activities from a rat sciatic nerve using a regeneration-based microchannel electrode array conduit^25^. The study featured recording of single unit spike patterns for gait cycle of walking rats up to 10 weeks post implantation^25^. However, for this type of regeneration-based microchannel devices, the number of fibers and the morphologies change after they are regrown into the microchannel^22^, which might compromise the integrity of investigation for the natural neurophysiology of VGVN and GI organs. However, we acknowledge that a thorough assessment of the long-term biological interaction between the Flex-µCh device and the VGVN might be necessary in the future to compare the long-term functionality with other types of peripheral nerve interface devices including the thick elastomer-based microchannel device^24^ and polyimide-based intraneural device^43^.

Microchannels in their traditional use provide isolated conduits for nerve fibers that enable high quality recording of SFAP activity, but they require relatively risky and cumbersome processes such as regeneration or teasing of nerve fibers to record neural signals. For the next generation microchannel devices, it might be necessary to implement a design that complements benefits from microchannel and probe array devices: The penetration capability from probe type could be integrated to the microchannel device to enable facile isolation of fibers to array of microchannels with recording electrodes. Overcoming the risk of damage to the nerve or nerve fibers will remain a challenge. Chronic recording stability and the longevity of the device after the implantation are also necessary future work after resolving the issues.

Although the Pt black coating significantly reduced the impedance of the recording electrode, we acknowledge that there are concerns regarding the mechanical robustness of the Pt black coating. However, we believe that the stability of the Pt black or any other type of surface engineering effort to lower the impedance could be enhanced with more research and development. For example, Kim and Nam incorporated polydopamine to Pt black coating to enhance mechanical stability^44^. This strategy can be potentially applied to our Flex-µCh device for a long-term recording study.

Going forward, investigation on the ultrastructure of the teased VGVN fibers by transmission electron microscopy (TEM) could tell us more about the relationship between the waveform shape of ECAP signals and fiber morphologies at each microchannel: The knowledge is critical to predict ECAP signals, which are results of constructive or destructive interferences between ECAPs from various group of fibers that are activated with different stimulus parameter combinations. We believe our Flex-µCh device would be a useful tool to investigate vastly unknown neurophysiology of VGVN and GI organs in the future as we successfully overcome remaining challenges.

## Conclusion

In this paper, we suggested a novel microchannel electrode array device implemented on a flexible thin-film substrate. The Flex-µCh device is designed to interface with rat VGVN, which is located at the esophagus close to stomach, in an area subject to mechanical motion due to organ motility. The thin-film platform minimizes volumetric footprint around VGVN, so that it can reduce trauma caused by mechanical interaction. The electrodes are coated with Pt-black, which reduces impedance by micro & nano scale rough morphology to ensure high quality recording of neural signals. We demonstrated surgical implantation of the device to rat VGVN and recording ECAP signals evoked from the cervical VNS. The electrode array that interfaced transected end of VGVN clearly recorded ECAP signals from unmyelinated C-fiber conduction velocity range with SNR up to 28.0 dB which could be regarded as a high-quality signal. A critical next step is to develop an advanced nerve teasing method to successfully tease rat VGVN, which has 100 µm of diameter, and isolate them into the microchannels to record patterns of neural signals with higher resolution.

### Supplementary information

Supplemental Material
