# Supplementary material for: A flexible, thin-film microchannel electrode array device for selective subdiaphragmatic vagus nerve recording
Source: Microsyst Nanoeng. 2024 Jan 23;10:16. doi: 10.1038/s41378-023-00637-6 (PMC10803373; doi:10.1038/s41378-023-00637-6)
Supplement: Supplementary file 1 — Supplemental Material [file 41378_2023_637_MOESM1_ESM.docx]

**Supplementary information**

**A flexible, thin-film microchannel electrode array device for selective subdiaphragmatic vagus nerve recording**

Jongcheon Lim^1,2,3^, Peter A. Zoss^1^, Terry L. Powley^1,4,5^, Hyowon Lee^1,2,3*^, and Matthew P. Ward^1,6*^

^1^ Weldon School of Biomedical Engineering, Purdue University, West Lafayette, IN, USA

^2^ Birck Nanotechnology Center, Purdue University, West Lafayette, IN, USA

^3^ Center for Implantable Devices, Purdue University, West Lafayette, IN, USA

^4^ Department of Psychological Sciences, Purdue University, West Lafayette, IN, USA

^5^ Purdue Institute of Integrative Neuroscience, Purdue University, West Lafayette, IN, USA

^6^ Indiana University School of Medicine, Indianapolis, IN, USA

*Co-corresponding Author E-mails: [mpward@purdue.edu](mailto:mpward@purdue.edu), [hwlee@purdue.edu](mailto:hwlee@purdue.edu)

**Supplementary experimental details**

*Transmission electron microscope (TEM)*

The TEM sample preparation procedure was adapted from previous research on ultrastructure of vagus nerve^1^ so that our research can be expanded to the ultrastructure study in the future. Flex-µCh device was embedded in Epon epoxy plastic by curing the resin for 48 hours at 37 ºC. The specimen was dissected into ultrathin sections (70-90 nm) using a 45º diamond ultramicrotome. The sectioned specimen was mounted on a formvar-coated single slot copper grid and analyzed with TEM (FEI Tecnai G2 20) at 80 kV.

*Sample preparation for the microscopic images of nerve fibers in the VN*

LCVN and VGVN were harvested from the rat after the euthanasia. The harvested nerves were treated with 2 mg mL^-1^ of collagenase solution in PBS (Type 4 collagenase, Worthington Biochemical Corporation, Lakewood, NJ, USA) for > 10 min to aid smooth dissociation of nerve fibers from collagenous tissue structure such as epineurium and perineurium^2^. After the treatment, the nerve samples were transferred to a glass slide substrate and a glass cover slip was placed on it before observing with an optical microscope (KH-8700, Hirox Co Ltd, Tokyo, Japan).

**Fig.S1.**


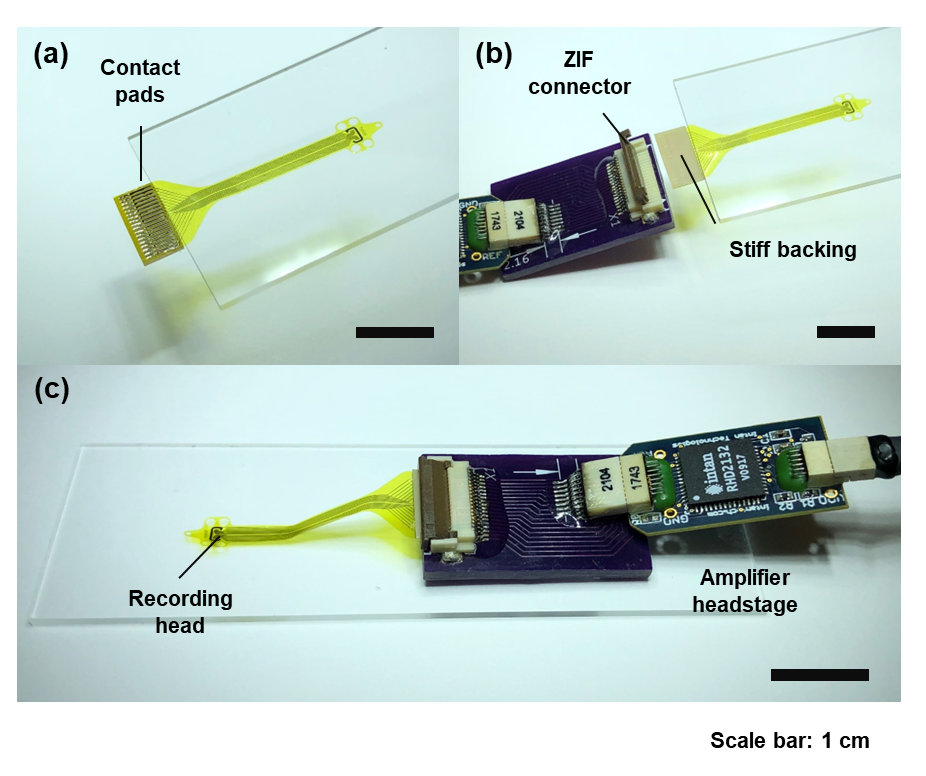


Fig.S1. Photographic images that illustrate the assembly of the device to the data acquisition system. (a) The flexible thin-film based microchannel electrode array device placed on a glass slide with a PEEK stiff backing attached to the backside of the contact pads. (b) The device was flipped to face the contact pads to the pins of the ZIF connector. (c) Device as connected to the amplifier headstage with the recording head facing the surface of the glass slide.

**Fig.S2.**


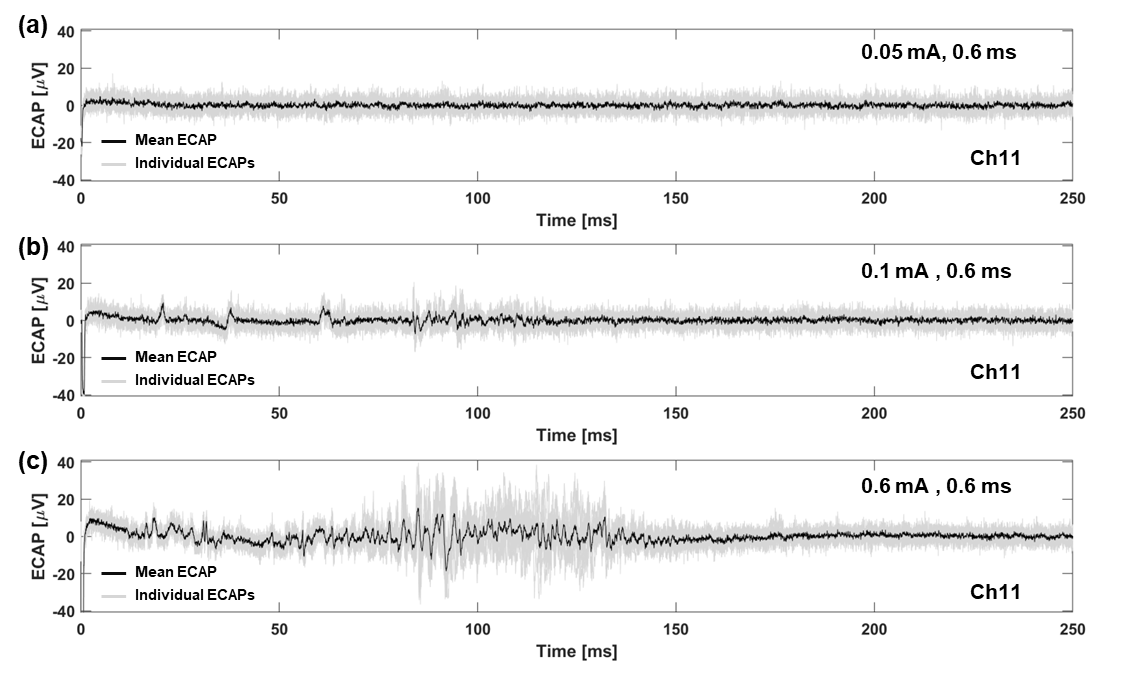


Fig.S2. ECAP signals from one of the electrodes where the strongest intensity of the signals are observed (Ch11 electrode). (a-c) Mean (solid black line, N=10) and individual (overlapped grey lines) ECAP signals with different pulse current amplitudes at pulse duration of 0.6 ms.

**Fig.S3.**


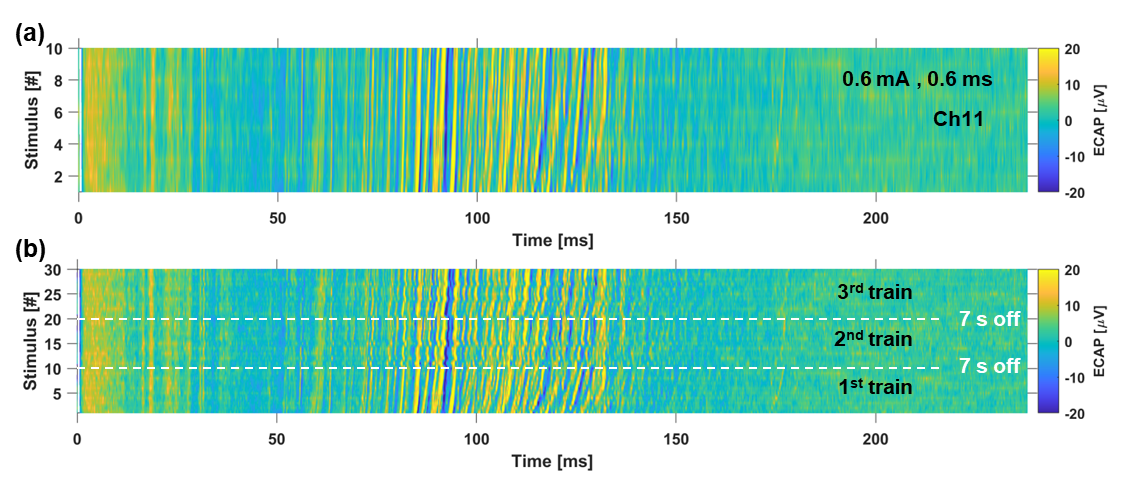


Fig.S3. Color plot of the ECAP at a fixed stimulus parameter (0.6 mA, 0.6 ms) with respect to the repetition of stimulus. Peaks get delayed to the later latency as the stimulus number in the repetition increases (a). With three consecutive stimulus trains applied with 7 s off time in between, the delayed peaks instantaneously recover toward original latency where the stimulus is paused (b).

**Supplementary references**

1 Havton, L. A. *et al.* Human organ donor-derived vagus nerve biopsies allow for well-preserved ultrastructure and high-resolution mapping of myelinated and unmyelinated fibers. *Scientific Reports* **11**, 23831 (2021).

2 Chen, L., Ilham, S., Guo, T., Emadi, S. & Feng, B. In vitro multichannel single-unit recordings of action potentials from the mouse sciatic nerve. *Biomedical physics & engineering express* **3**, 045020 (2017).
